# Reaction N(^2^D) + CH_2_CCH_2_ (Allene): An Experimental and Theoretical Investigation and Implications for the Photochemical Models of Titan

**DOI:** 10.1021/acsearthspacechem.2c00183

**Published:** 2022-09-29

**Authors:** Gianmarco Vanuzzo, Luca Mancini, Giacomo Pannacci, Pengxiao Liang, Demian Marchione, Pedro Recio, Yuxin Tan, Marzio Rosi, Dimitrios Skouteris, Piergiorgio Casavecchia, Nadia Balucani, Kevin M. Hickson, Jean-Christophe Loison, Michel Dobrijevic

**Affiliations:** † Dipartimento di Chimica, Biologia e Biotecnologie, Università degli Studi di Perugia, 06123 Perugia, Italy; ∥ Dipartimento di Ingegneria Civile e Ambientale, Università degli Studi di Perugia, 06100 Perugia, Italy; ⊥ Master-Tec SrL, Via Sicilia, 41, 06128 Perugia, Italy; # Université de Bordeaux, Institut des Sciences Moléculaires, UMR 5255, F-33400 Talence, France; ∇ CNRS, Institut des Sciences Moléculaires, UMR 5255, F-33400 Talence, France; ○ Laboratoire d’Astrophysique de Bordeaux, Université de Bordeaux, CNRS, B18N, allée Geoffroy Saint-Hilaire, F-33615 Pessac, France

**Keywords:** atmospheric chemistry of Titan, prebiotic chemistry, reactivity of electronically excited species, nitrogen chemistry, formation of N-containing organic molecules

## Abstract

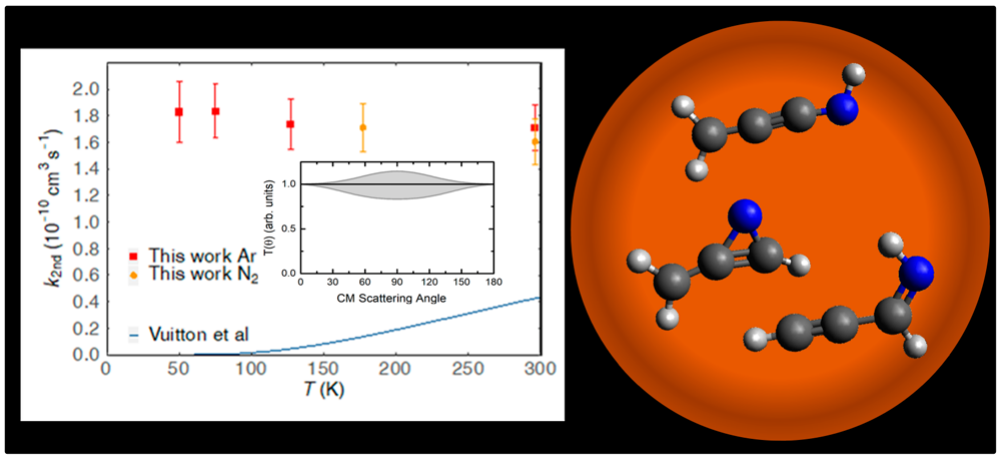

We report on a combined experimental and theoretical investigation of the N(^2^D) + CH_2_CCH_2_ (allene) reaction of relevance in the atmospheric chemistry of Titan. Experimentally, the reaction was investigated (i) under single-collision conditions by the crossed molecular beams (CMB) scattering method with mass spectrometric detection and time-of-flight analysis at the collision energy (*E*_c_) of 33 kJ/mol to determine the primary products and the reaction micromechanism and (ii) in a continuous supersonic flow reactor to determine the rate constant as a function of temperature from 50 to 296 K. Theoretically, electronic structure calculations of the doublet C_3_H_4_N potential energy surface (PES) were performed to assist the interpretation of the experimental results and characterize the overall reaction mechanism. The reaction is found to proceed via barrierless addition of N(^2^D) to one of the two equivalent carbon–carbon double bonds of CH_2_CCH_2_, followed by the formation of several cyclic and linear isomeric C_3_H_4_N intermediates that can undergo unimolecular decomposition to bimolecular products with elimination of H, CH_3_, HCN, HNC, and CN. The kinetic experiments confirm the barrierless nature of the reaction through the measurement of rate constants close to the gas-kinetic rate at all temperatures. Statistical estimates of product branching fractions (BFs) on the theoretical PES were carried out under the conditions of the CMB experiments at room temperature and at temperatures (94 and 175 K) relevant for Titan. Up to 14 competing product channels were statistically predicted with the main ones at *E*_c_ = 33 kJ/mol being formation of *cyclic*-CH_2_C(N)CH + H (BF = 87.0%) followed by CHCCHNH + H (BF = 10.5%) and CH_2_CCNH + H (BF = 1.4%) the other 11 possible channels being negligible (BFs ranging from 0 to 0.5%). BFs under the other conditions are essentially unchanged. Experimental dynamical information could only be obtained on the overall H-displacement channel, while other possible channels could not be confirmed within the sensitivity of the method. This is also in line with theoretical predictions as the other possible channels are predicted to be negligible, including the HCN/HNC + C_2_H_3_ (vinyl) channels (overall BF < 1%). The dynamics and product distributions are dramatically different with respect to those observed in the isomeric reaction N(^2^D) + CH_3_CCH (propyne), where at a similar *E*_c_ the main product channels are CH_2_NH (methanimine) + C_2_H (BF = 41%), c-C(N)CH + CH_3_ (BF = 32%), and CH_2_CHCN (vinyl cyanide) + H (BF = 12%). Rate coefficients (the recommended value is 1.7 (±0.2) × 10^–10^ cm^3^ s^–1^ over the 50–300 K range) and BFs have been used in a photochemical model of Titan’s atmosphere to simulate the effect of the title reaction on the species abundance (including any new products formed) as a function of the altitude.

## 1 Introduction

The study of other planets (or moons) of the solar system can be of great help in understanding prebiotic chemistry and the initial chemical evolution of Earth, where the presence of a biosphere and plate tectonics have drastically changed the primitive conditions that harbored life emergence.^1^ In this respect, Titan (the massive moon of Saturn) has attracted a lot of attention^2^ because its atmosphere is mainly composed of molecular nitrogen, like the terrestrial one, but sustains a very complex organic chemistry starting with methane, the second most abundant component (ca. 2% in the stratosphere and up to 5% close to the surface of the moon).^3^ Among the organic species detected in trace amounts, the presence of nitriles and other N-containing organic species clearly indicates that active forms of nitrogen are at play.^4,5^ In the upper atmosphere of Titan, chemistry is initiated by ionization and dissociation of the two main components (N_2_ and CH_4_), both induced by either VUV photons or collisions with energetic particles such as the electrons from the magnetosphere of Saturn.^4,5^ These processes represent the starting point of a complex network of chemical reactions. Atomic nitrogen can be formed by N_2_ EUV photodissociation or by dissociative electron impact as well as other processes like N_2_^+^ dissociative recombination. N atoms are produced not only in their ground ^4^S state but also in their first electronically excited metastable states.^6^ Among them, the ^2^D_3/2,5/2_ state is of great relevance because its radiative lifetime is very long (the transition to the ground state is doubly forbidden) and is much more reactive than the ground ^4^S state.^6^ For this reason, N(^2^D) reactions have been considered to play an important role since the first photochemical model of Titan developed by Yung et al. in 1984,^7^ where N(^2^D) reactions with methane^7^ and acetylene^8^ were included with estimated rate coefficients and products.

Because of the difficulty in producing N(^2^D) in a controlled manner, until recently only fragmentary information was available from laboratory experiments on the reactions of N(^2^D). After detailed investigation by means of the crossed molecular beam (CMB) method, supported by electronic structure calculations of the underlying potential energy surface (PES), now we know that N(^2^D) has a complex chemical behavior, being able to insert into sigma bonds or to add to multiple bonds.^9−20^ When reacting with hydrocarbons, molecular products possessing a novel C–N bond are formed.^9−14,18−20^ Recent kinetic experiments performed with the CRESU technique at the relevant temperature for the conditions of the upper atmosphere of Titan have revealed that the rate constants for several important N(^2^D) reactions are considerably larger^21,22^ or smaller^23^ than those determined by previous experiments above 200 K.^24−26^ The inclusion of these data in updated versions of a photochemical model of the atmosphere of Titan have demonstrated the importance of this approach in making the model more accurate.^21−23^

Methylacetylene (propyne) and allene (propadiene) are two structural isomers of gross formula C_3_H_4_ that are formed in the upper atmosphere of Titan. Both are predicted to be present by all photochemical models in similar amounts because their main formation mechanism is considered to be the reaction H + C_3_H_5_, producing both isomers with the same yield. However, while methylacetylene was first detected during the Voyager mission,^27−29^ attempts to detect allene were unsuccessful^30−32^ until the recent unambiguous detection by Lombardo et al.^33^ by means of a Texas Echelle Cross Echelle Spectrograph (TEXES) mounted on the NASA Infrared Telescope Facility. Allene has an abundance of 6.9 (±0.8) × 10^–10^ at an altitude of 175 km and is less abundant than methylacetylene by a factor of 8.2 (±1.1) at 150 km if a vertically increasing profile is assumed.^33^

The reactions between atomic nitrogen in its ^2^D state and both methylacetylene and allene have already been included in photochemical models with estimated rate coefficients and product branching fractions (BFs).^4,5^ In the case of the N(^2^D) + CH_2_CCH_2_ reaction, by analogy with similar systems, Loison et al.^4^ suggested that the main reaction channel is that leading to vinyl cyanide (cyanoethylene) + H with a global rate coefficient of 2.3 × 10^–10^ × exp(−503/*T*). However, according to the present B3LYP/CCSD(T) calculations, there are many additional open reactive channels correlating with the reactants
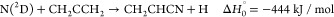
1a
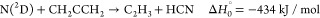
1b
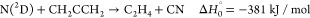
1c
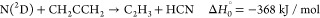
1d
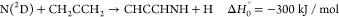
1e
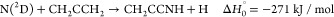
1f
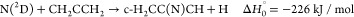
1g
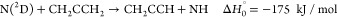
1h
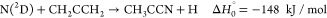
1i
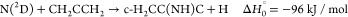
1j
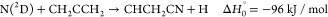
1k
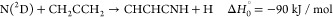
1l
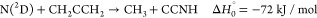
1m
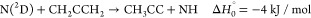
1nwhere the enthalpies of reactions reported are those calculated in the present work at the CCSD(T) level (see section 3.1). There are eight H-displacement channels leading to eight different C_3_H_3_N isomeric species, two NH formation channels, and four C–C bond-breaking channels leading to HCN, HNC, CN, and CH_3_ formation.

In addition, an H_2_-elimination channel is also possible through a roaming mechanism (see section 5.1)
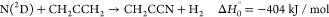
1owith the enthalpy of reaction obtained at the same level of calculations as the other channels.

In this manuscript, we report on a combined experimental and theoretical investigation of the reaction N(^2^D) + CH_2_CCH_2_. More specifically, we employed the CMB technique to explore the nature of the primary products and their BFs and the CRESU technique to measure the global rate coefficient at temperatures of interest for Titan. In addition, we performed dedicated electronic structure calculations of the underlying PES and RRKM (Rice–Ramsperger–Kassel–Marcus) estimates of the product BFs. The information so obtained are used in a photochemical model of Titan’s atmosphere to simulate the effect of this reaction on the species abundance (including any new products formed) as a function of altitude. A comparison with the reaction mechanism of the reaction involving the methylacetylene isomer will also be presented to highlight similarities and differences.

## 2 Experimental Section

### 2.1 Crossed Molecular Beam Experiments

The scattering experiments were carried out using an improved version of the CMB apparatus described previously.^34−37^ Briefly, two supersonic beams of the reactants are crossed at a specific angle (90°) in a large scattering chamber kept in the low 10^–6^ mbar range in operating conditions to ensure single-collision conditions. The species of each beam are characterized by a well-defined velocity and direction and are made to collide only with the atoms/molecules of the other beam, allowing us to observe the consequences of well-defined reactive molecular collisions. The detection system consists of a tunable electron impact ionizer, a quadrupole mass filter, and a Daly detector. The ionizer is located in the innermost region of a triply differentially pumped ultrahigh-vacuum chamber, which is maintained in the 10^–11^ mbar pressure range in operating conditions by extensive turbo- and cryopumping. The whole detector unit can be rotated in the collision plane around an axis passing through the collision center, and the velocities of both reactants and products are derived from single-shot and pseudorandom, respectively, time-of-flight (TOF) measurements.

A supersonic beam of N atoms was produced by means of a radio frequency (rf) discharge beam source described in refs (38) and (39). We operated by discharging 250 W of rf power on a dilute (2.5%) mixture of N_2_ in He (stagnation pressure of 125 mbar), expanded through a 0.48 mm diameter quartz nozzle followed by a boron nitride skimmer (diameter of 0.8 mm) placed at a distance of about 6 mm from the front of the nozzle. The peak velocity and speed ratio were 2354 m/s and 5.9, respectively. A high dissociation of molecular nitrogen (about 60%) is achieved. Nitrogen atoms are produced in a distribution of electronic states as shown in a previous characterization by means of Stern–Gerlach magnetic analysis.^39^ Seventy-two percent of the N atoms were found to be produced in the ground ^4^S state, while 21% and 7% are produced in the ^2^D and ^2^P excited state, respectively. Under the present experimental conditions, the use of a beam also containing nitrogen atoms in the electronic ^4^S and ^2^P states is not a problem since the rate coefficients for reactions between N(^4^S) and unsaturated hydrocarbons are extremely small, while N(^2^P) is known to mostly undergo physical quenching.^6^ We expect a similar situation also in the case of the title reaction. Indeed, according to the present theoretical characterization of the title reaction, the reactivity of the N(^4^S) state is not expected to be significant as we could not locate any addition intermediate in low-energy quartet states, while the H-abstraction channel is endothermic by 55 kJ/mol at the present level of calculations (and, therefore, it is not accessible under the conditions of our experiments or under the condition of the atmosphere of Titan).

A supersonic beam of allene was generated by expanding 400 mbar of neat allene through a 100 μm diameter stainless-steel nozzle kept at room temperature. A collimating stainless-steel skimmer of 0.8 mm diameter was placed 7 mm from the front of the nozzle. The peak velocity and speed ratio were 696 m/s and 4.5, respectively.

The angular divergence, which is defined by the collimating slits placed after the skimmers, is 2.4° for the N beam and 3.8° for the allene beam. The detector has a nominal angular resolution for a point collision zone of 1.1°.

The resulting collision energy is 33 kJ/mol, while the angle of the center-of-mass (CM) velocity vector in the laboratory (LAB) reference frame with respect to the velocity vector associated with the atomic nitrogen beam is Θ_CM_ = 40.7°. The product angular distribution, N(Θ), was recorded by means of a tuning-fork chopper (for background subtraction) mounted between the nozzle and the skimmer defining the allene beam (modulation frequency of 160 Hz). The velocity distributions of the products were measured by the pseudorandom chopping technique using four 127-bit open-closed sequences based on the cross-correlation method. High time resolution was obtained by spinning the TOF disk, located before the entrance slit of the detector, at 328 Hz, corresponding to a dwell time of 6 μs/ch. The flight length was 24.3 cm.

The measurements have been carried out in the LAB reference frame. However, in order to obtain quantitative information on the scattering process, it was necessary to move from the LAB to the CM frame; in this way, it is possible to derive the CM product flux distribution *I*_CM_(θ, *E*′_T_), i.e., the double-differential cross section. This can be factorized into two independent functions: a function depending only on the scattering angle, *T*(θ), and a function depending on the velocity, *P*(*u),* or translational energy, *P*(*E*′_T_), of the products.^35,36^ It is important to notice that because of the finite resolution of the experimental conditions, such as the angular and velocity spread of the reactant beams and the angular resolution of the detector, the conversion from the LAB to the CM reference system is not single valued. Therefore, the analysis of the LAB data have been performed by *forward convoluting* tentative CM distributions over the experimental conditions. In other words, the CM angular, *T*(θ), and translation energy, *P*(*E*′_T_), distributions are assumed, averaged, and transformed into the LAB frame for comparison with the experimental distributions. The procedure is repeated until a satisfactory fit of the experimental data is achieved.^34^

### 2.2 Kinetics Experiments

All of the kinetic measurements reported here were conducted using a continuous supersonic flow (Laval nozzle) reactor, whose main features have been described in detail previously.^40,41^ The original apparatus has been modified over the years to allow both ground state and excited state atomic radicals such as C(^3^P),^42,43^ H(^2^S),^44,45^ O(^1^D),^46,47^ and recently, N(^2^D)^21−23,48^ to be detected in the vacuum ultraviolet (VUV) wavelength range. To perform kinetic measurements over a range of low temperatures, three different Laval nozzles were utilized during this investigation, allowing four different temperatures (50, 75, 127, and 177 K) to be accessed (one nozzle was used with two carrier gases), in addition to room temperature (296 K) in the absence of a nozzle and at a significantly reduced flow velocity. As previous measurements of N(^2^D) quenching have shown that relaxation is slow with both Ar^49^ and N_2_,^50^ it was possible to use both of these as carrier gases. The flow characteristics of the nozzles used during this study are listed in Table 1 of Nuñez-Reyes et al.^21^ N(^2^D) atoms were produced indirectly during this study as a product of reaction 2a
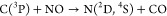
2a
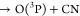
2bin common with previous work^21−23,48^ due to the lack of precursor molecules to produce this species photolytically in an appropriate wavelength range. The yield of total atomic nitrogen (N(^2^D) + N(^4^S)) has been estimated to be approximately three times greater than the yield of ground state atomic oxygen at room temperature.^51^ Ground state atomic carbon, C(^3^P), was produced in situ by the pulsed laser photolysis of carbon tetrabromide (CBr_4_) at 266 nm. CBr_4_ was introduced into the flow by passing a small flow of the carrier gas over solid CBr_4_ held in a separate container at a known pressure and room temperature. CBr_4_ concentrations were estimated to be lower than 4 × 10^13^ cm^–3^ based on its saturated vapor pressure, while NO concentrations were in the range 3.0–6.4 × 10^14^ cm^–3^.

In addition to C(^3^P), C(^1^D) atoms were also generated during CBr_4_ photodissociation with a C(^1^D)/C(^3^P) ratio measured in earlier work of 0.1–0.15.^42^ C(1D) atoms are expected to react rapidly with NO^52^ to form similar products to the ground state reaction (reactions 2a and 2b) and/or be quenched rapidly to the ground state when N_2_ is used as the carrier gas.^53^ The photolysis laser beam diameter was reduced from 12 to 5 mm using an afocal telescope, allowing significantly higher pulse energies (30–40 mJ) to be used than in previous kinetic studies (20–25 mJ). As larger C(^3^P) (and N(^2^D)) concentrations were generated, a significant improvement in the signal-to-noise ratio was observed.

N(^2^D) atoms were detected directly during this study by pulsed laser-induced fluorescence at 116.745 nm through the 2s^2^2p^3 2^D°–2s^2^2p^2^(^3^P)3d ^2^F electronic transition. The procedure used to generate tunable coherent radiation around this wavelength by third-harmonic generation of a monochromatic UV source focused into a cell containing rare gas has been described in detail in previous work.^48^ Upon exiting the cell, the VUV probe beam was collimated by a MgF_2_ lens and directed into the reactor through a 75 cm long side arm containing a series of circular diaphragms to trap the divergent UV beam. The side arm itself was attached to the reactor at the level of the observation axis, so that the VUV beam crossed the cold supersonic flow at right angles. In this way, it was also perpendicular to the detector. This arrangement ensured that only a tiny fraction of the residual UV light reached the detector. Fluorescence emission from excited N(^2^D) atoms in the flow, on resonance with the probe laser, was detected by a solar blind photomultiplier tube (PMT) which was protected from reactive gases in the chamber by a LiF window. A LiF lens placed between the window and the PMT focused the emitted light onto the PMT photocathode. As atmospheric O_2_ possesses numerous absorption features in this region of the electromagnetic spectrum, the zone between the LiF window and the PMT was maintained under vacuum. In contrast to previous work, the output of the PMT was fed directly into a boxcar integrator without the need for prior amplification. Nevertheless, the first 5 μs following the photolysis laser pulse remained unexploitable (compared to 15 μs in previous work when an amplifier was used) due to scattering of the photolysis laser beam by the precursor CBr_4_ molecules in the supersonic flow. Typically, between 70 and 100 time points (including approximately 15 time points to establish the baseline) were recorded for each kinetic profile with 30 laser shots averaged at each time point. All gas flows (Messer Ar 99.999%, N_2_ 99.995%, Linde Xe 99.999%, Sigma-Aldrich CH_2_CCH_2_ >95%, Air Liquide NO 99.9%) were controlled by calibrated mass flow controllers, allowing the coreagent NO and CH_2_CCH_2_ concentrations to be determined accurately.

## 3 Computational Details

### 3.1 Electronic Structure Calculations

The N(^2^D) + CH_2_CCH_2_ reaction has been analyzed by considering the lowest doublet electronic state of the C_3_H_4_N system. The potential energy surface has been characterized through optimization of the most stable stationary points at the B3LYP^54,55^ level of theory in conjunction with the correlation-consistent valence-polarized set aug-cc-pVTZ.^56−58^ Harmonic vibrational frequencies have been computed at the same level of theory in order to check the nature of the stationary points, i.e., minimum if all frequencies are real, saddle point if there is one and only one imaginary frequency. Intrinsic reaction coordinate (IRC) calculations have been performed to assign the nature of each saddle point.^59,60^ More accurate values of energy of all of the stationary points have been calculated at the higher level of calculation CCSD(T)^61−63^ with the same basis set aug-cc-pVT. The zero-point energy (ZPE) correction, computed using the scaled harmonic vibrational frequencies evaluated at the B3LYP/aug-cc-pVTZ level, has been added to both the B3LYP and the CCSD(T) energies to correct them at 0 K. The energy of N(^2^D) has been evaluated by adding the experimental^64^ separation N(^4^S)–N(^2^D) of 230.0 kJ/mol to the energy of N(^4^S) at all levels of calculation. All calculations have been performed using Gaussian 09,^65^ while the analysis of the vibrational frequencies has been carried out using AVOGADRO.^66,67^

### 3.2 RRKM Calculations

RRKM calculations for the N(^2^D) + CH_2_CCH_2_ reaction have been performed using a code developed in our group for this purpose.^11−13^ As suggested by the RRKM scheme,^68^ the microcanonical rate constant, *k*(*E*), for a specific reaction at a specific total energy is given by the expression
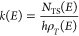
where *N*_TS_(*E*) is the sum of states of the transition state at energy *E*, ρ_*T*_(*E*) is the reactant density of states at energy *E*, and *h* is Planck’s constant. The partition function has been used to perform an inverse Laplace transform in order to evaluate the rotational densities of states both for the reactants and for the transition states. Subsequently, the rotational densities of states were convoluted with the corresponding vibrational ones using a direct count algorithm. Finally, the sum of states has been obtained by integrating the density of states with respect to the energy. Where possible, tunneling (as well as quantum reflection) has been considered by using the corresponding imaginary frequency of the transition state and calculating the tunneling probability for the corresponding Eckart barrier. For barrierless dissociation channels, the variational RRKM approach is normally used.^69^ In this case, however, that approach could not be employed because of some difficulties in the electronic structure calculations of the intermediate points. Considering that the channels of interest are characterized by energetically monotonic exit paths, the transition state has been assumed as the products at infinite separation. The way we avoid problems arising from the different number of degrees of freedom between the reactants and the transition state is by not including the 2D part of the overall rotation in the RRKM treatment of the reactants (leaving only the “prolate” 1D contribution).

After the calculation of all microcanonical rate constants, a Markov (stochastic) matrix was set up for all intermediates and final channels to derive the product branching fractions for the overall reaction. *k*(*E*) is subsequently Boltzmann averaged for each temperature of interest to yield *k*(*T*).

## 4 Experimental Results

### 4.1 CMB Experiments

Preliminary measurements were made at different mass-to-charge ratios (*m*/*z*). The signal was observed at the following: (1) *m*/*z* = 53 (C_3_H_3_N^+^), which corresponds to the parent ion of molecular products in the H-displacement channels; (2) *m*/*z* = 52 (C_3_H_2_N^+^), which corresponds either to the parent ion of the molecular product associated with the H_2_-elimination channel or the −1 daughter ion associated with the H-displacement channels; (3) *m*/*z* = 51 (C_3_HN^+^) and 50 (C_3_N^+^), which correspond to the daughter ions of cases 1 and 2. In this range of masses, the signal at *m*/*z* = 51 was found to be the most intense one with the highest signal-to-noise (S/N) ratio (with a 50 s counting time, the S/N ratios were 46, 54, 82, and 30, respectively). During data analysis, no features of the measured distributions pointed to the presence of an H_2_-elimination channel, so the signal recorded at *m*/*z* between 50 and 53 can be attributed to the H-displacement channels 1a, 1e–1g, and 1i–1l. No signal was detected at *m*/*z* = 27, which rules out (within our sensitivity, i.e., BR ≤ 5%) channels 1b and 1d, leading to HCN and HNC formation, respectively.

We also attempted to measure reactive scattering distributions at *m*/*z* = 26 and 28 to characterize channel 1c leading to CN + C_2_H_4_. Unfortunately, we have not been able to verify if a small reactive scattering signal is present at those masses because of (a) a strong interfering signal at *m*/*z* = 28 associated with the elastic scattering of undissociated molecular nitrogen from the primary beam and (b) an interfering signal at *m*/*z* = 26 also coming from the primary beam. This is probably caused by the presence in the gas line of traces of CO_2_ which dissociates and reacts with N/N_2_ forming CN in the plasma produced by the radio frequency discharge.

Finally, since a CH_3_-loss channel is possibly open (channel 1m) and considering that the cofragment distributions at *m*/*z* = 39 (C_2_NH^+^) could not be measured because of an intense elastic signal associated with the dissociative ionization of allene, we tried to record a TOF distribution at *m*/*z* = 15 (CH_3_^+^) by using the soft-ionization approach (17 eV and an emission current of 1.50 mA). After an accumulation time of 45 min, no signal was observed at Θ = 40°. This ruled out, within our sensitivity, the occurrence of channel 1m.

The full set of final data, that is the LAB angular distributions and TOF spectra at Θ = 24°, 32°, 40°, and 48° (counting times ranging from 2 to 3 h per angle depending on the signal intensity), were recorded at *m*/*z* = 51.

**Figure 1:**
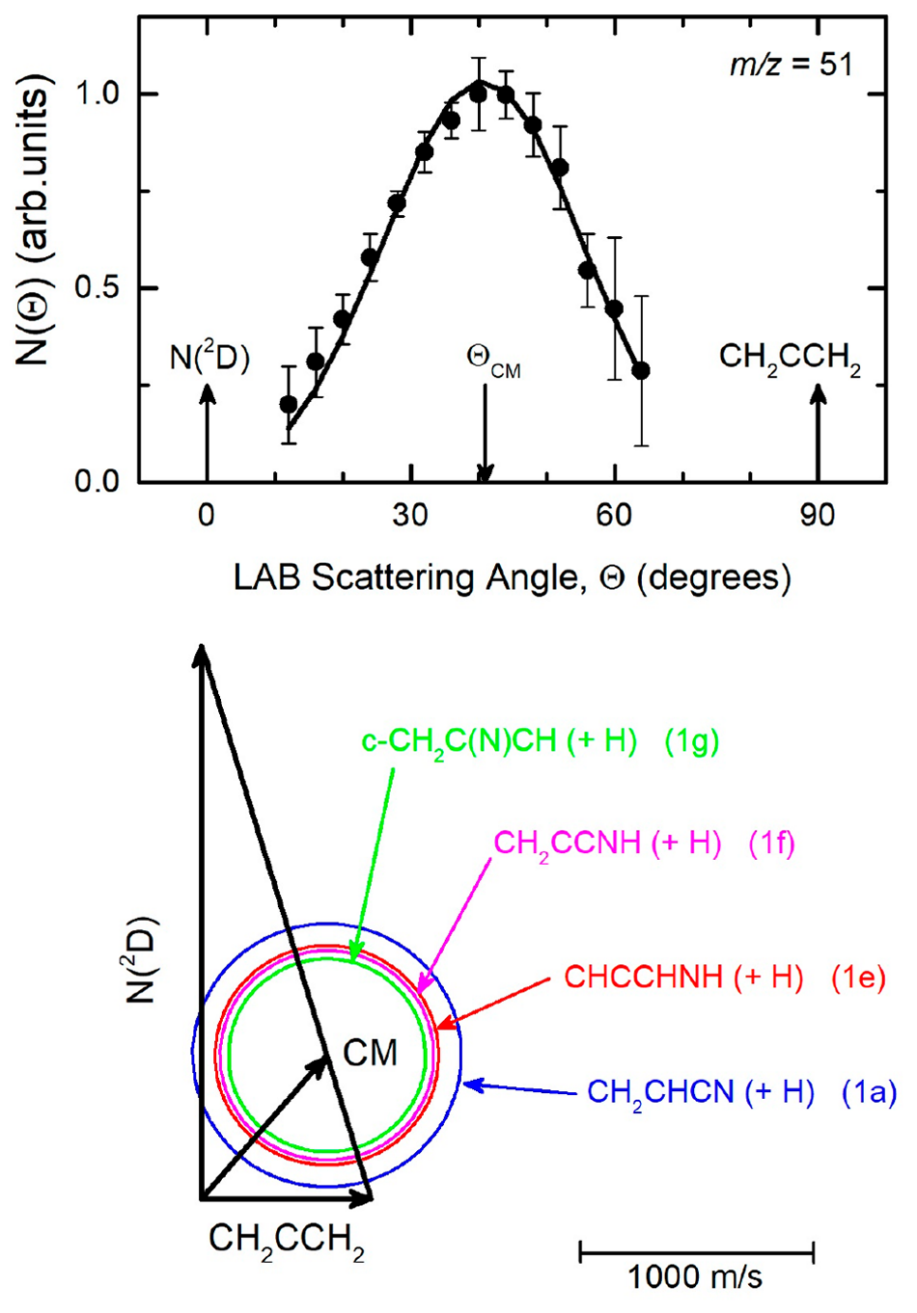
(Top) LAB angular distribution at *m*/*z* = 51 for the N(^2^D) + allene reaction at E_c_ = 33 kJ/mol. The solid line represents the calculated distribution when using the best-fit CM functions of Figure 3. (Bottom) Velocity vector (Newton) diagram of the CMB experiment. The radius of each circle represents the maximum speed that the indicated product can attain in the center-of-mass reference frame if all available energy is channeled into product recoil energy.

To better illustrate the CMB experimental results and discuss the dynamics of the various reaction channels, it is useful to observe the velocity vector (so-called Newton) diagram shown in Figure 1 (bottom), which describes the kinematics of the experiment. The circles are drawn assuming that all of the available energy is converted into product translational energy, and therefore, they delimit the maximum speed that the various indicated products can assume in the CM frame. Only the products associated with the most exothermic H-displacement channels 1a, 1e, 1f, and 1g are shown because we expect a negligible contribution from the other isomers (see Discussion).

**Figure 2:**
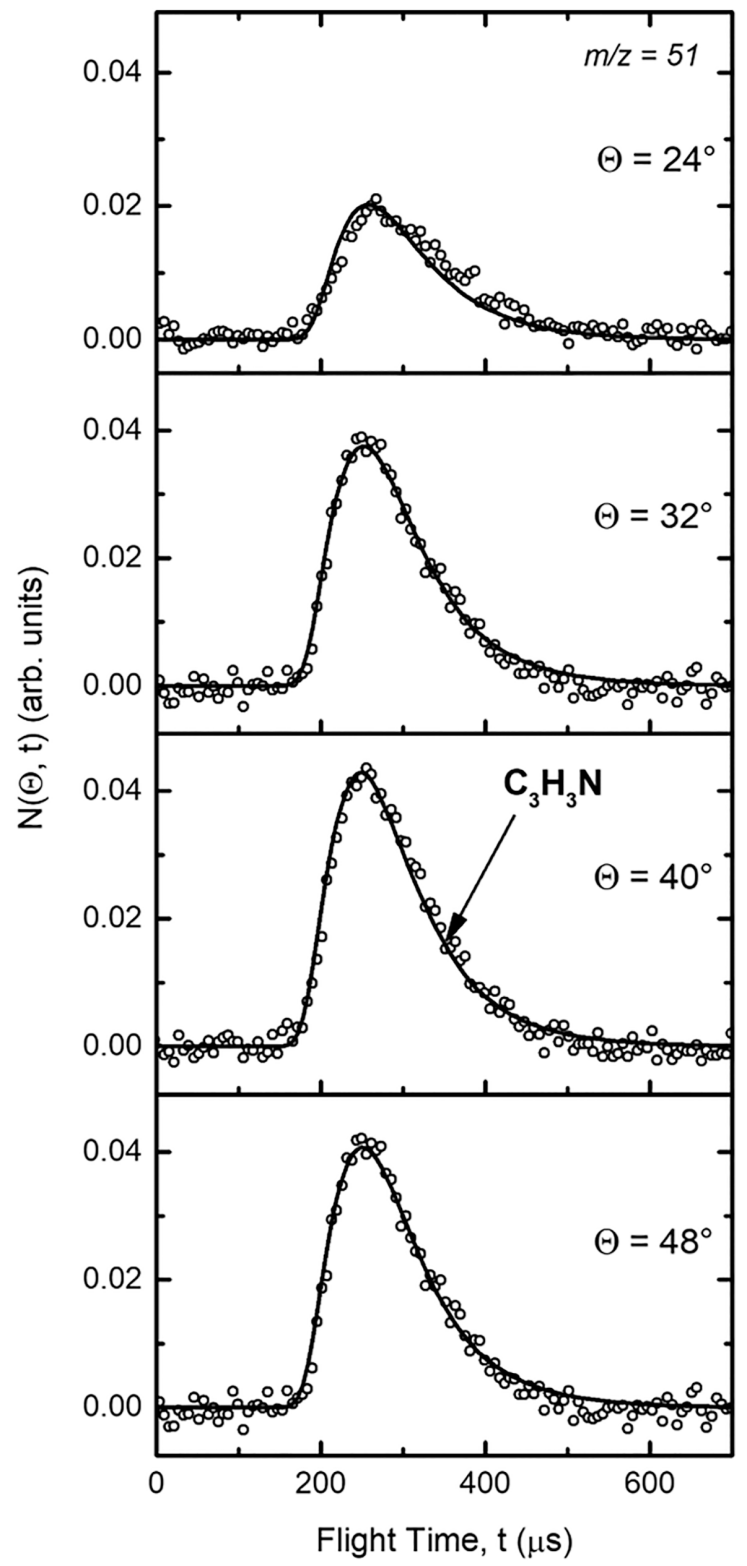
Time-of-flight distributions for *m*/*z* = 51 at angles Θ = 24°, 32°, 40°, and 48°. Open circles: experimental data. Solid lines: calculated distributions when using the best-fit CM functions of Figure 3.

The LAB angular distribution recorded at *m*/*z* = 51 is shown in Figure 1 (top). It is characterized by a bell shape and peaks at Θ_CM_. The relatively small extension confirms that the products are kinematically constrained in small Newton circles around the CM angle, in line with those shown in Figure 1 (bottom). The product TOF spectra at four selected LAB angles are displayed in Figure 2. As can be seen, the TOF spectra are characterized by a single peak, centered around 250 μs.

**Figure 3:**
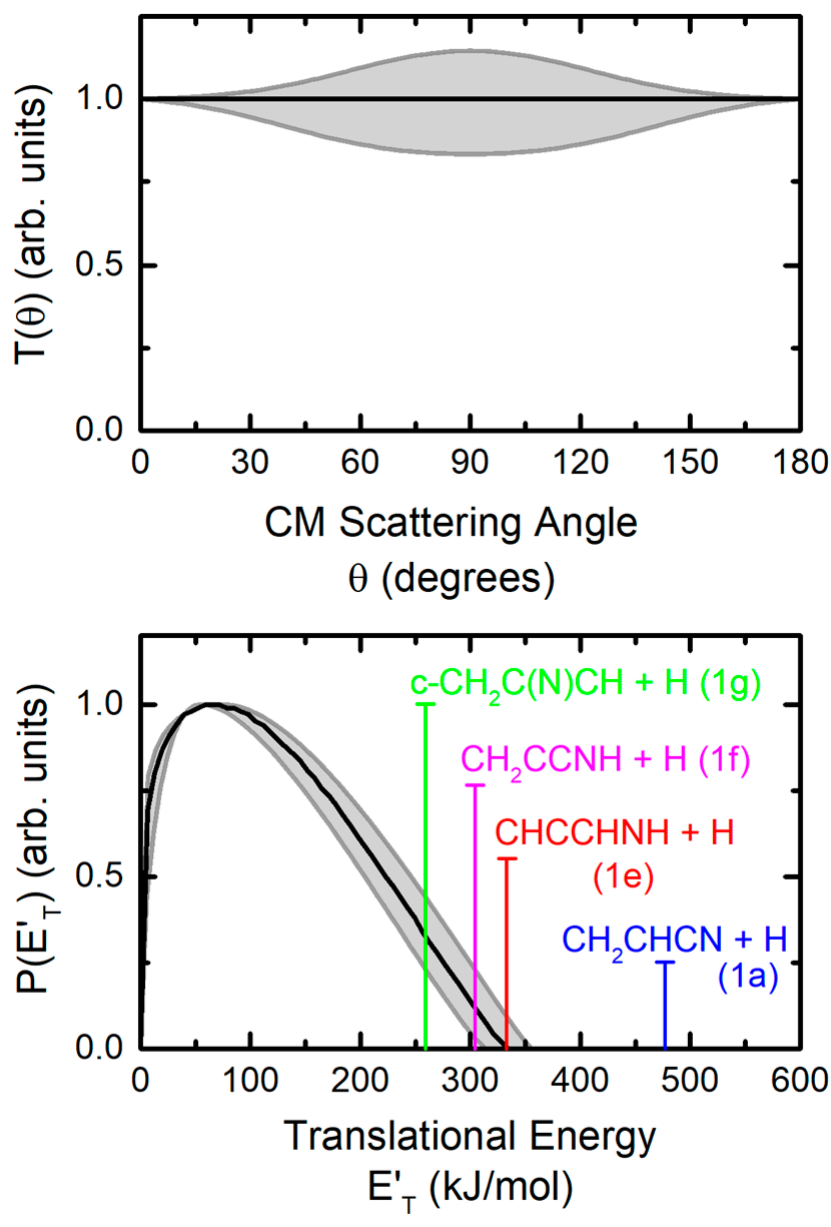
Best-fit angular (top) and translational energy (bottom) distributions, *T*(θ) and *P*(*E*′_T_), in the center of mass reference system for the N(^2^D) + allene reaction. Shaded areas represent the error bars. Arrows in the graph of *P*(*E*′_T_) indicate the total energy (*E*_c_ – Δ*H*^0^_0_) associated with the formation of the four indicated isomers with formula C_3_H_3_N.

The best-fit CM functions are shown in Figure 3. As can be seen, the best-fit CM angular distribution is isotropic. In addition, the functions that allow an acceptable fit of the data (delimited by the shaded areas in Figure 3, top) are all backward–forward symmetric, indicating that the title reaction proceeds through the formation of a long-lived complex.^70^ We recall that in this case, the collision complex survives several rotational periods, losing memory of the initial approach directions of the reactants. At the same time, its lifetime is long enough to allow the energy available to the system to be statistically distributed among the various degrees of freedom. This is an important indication as it sustains the applicability of an RRKM approach to derive the product branching fractions.

The shape of the *P*(*E*′_T_) reveals the extent of energy release, which give us a criterion, according to the energy conservation rule,^70^ to establish which products of general formula C_3_H_3_N are compatible with the experimental distributions. In our experimental conditions, the translational energy distribution has a maximum at about 60 kJ/mol and extends up to about 335(±20) kJ/mol. The average product translational energy, defined as ⟨*E*′_T_⟩ = ∑ *P*(*E*′_T_)*E*′_T_/∑*P*(*E*′_T_), is about 116 kJ/mol and corresponds to an average fraction, ⟨*f*′_T_⟩, of 0.35 of the total available energy (*E*_tot_ = *E*_c_ – Δ*H*°_0_) for the most exothermic H-displacement channel that was found to contribute significantly to the overall yield, namely, channel 1e (CHCCHNH + H) (see section 5.2). Given the similar enthalpy changes associated with the most exothermic H-displacement channels 1a, 1e, 1f, and 1g and the expected similar reaction mechanisms, we have not been able to disentangle the contributions of each channel to the recorded signal. We must rely on electronic structure and RRKM calculations to derive the product branching fractions (see sections 5.1 and 5.2).

### 4.2 Kinetic Results

**Figure 4:**
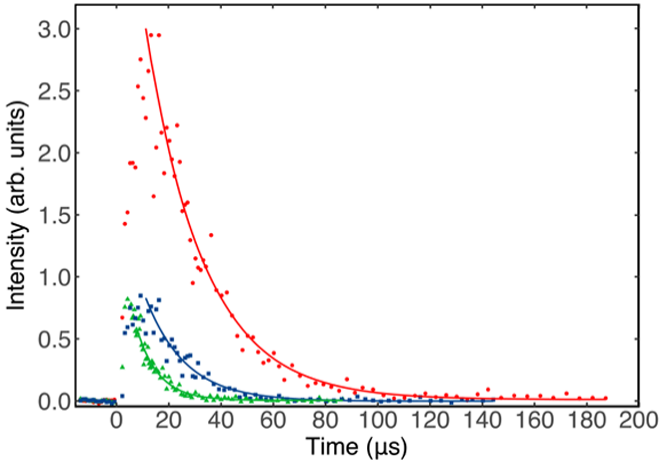
N(^2^D) fluorescence emission intensity as a function of time between photolysis and probe lasers, recorded at 127 K: (solid red circles) without CH_2_CCH_2_; (solid blue squares) [CH_2_CCH_2_] = 1.1 × 10^14^ cm^–3^; (solid green triangles) [CH_2_CCH_2_] = 4.3 × 10^14^ cm^–3^. [NO] = 4.7 × 10^14^ cm^–3^ for all traces. Solid lines represent nonlinear Levenberg–Marquardt fits to the data using expression 4.

The analysis of the kinetic data was simplified by employing the pseudo-first-order approximation by using large excess concentrations of the coreagents [NO] and [CH_2_CCH_2_] with respect to the minor reagents C(^3^P) and N(^2^D). Under these conditions, the N(^2^D) fluorescence signal should follow a temporal profile with a biexponential form given by
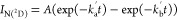
3where *A* is the theoretical maximum signal amplitude when the first term in expression 3 is equal to zero, *k*_**a**_^**′**^ is the pseudo-first-order rate constant for N(^2^D) loss, *k*_**b**_^**′**^ is the pseudo-first-order rate constant for N(^2^D) formation, and *t* is time. Although more early time points were exploitable in this work than in previous studies of N(^2^D) reactions as explained above, an analysis employing a single-exponential function
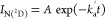
4was still employed due to the difficulty of performing accurate fits during the rising part of the temporal profiles. The fitting procedure was applied only to those data points obeying a single-exponential decay law, essentially excluding data within the first 10–20 μs following the photolysis laser pulse. Some typical decay traces recorded at 127 K are shown in Figure 4.

In common with earlier work, it is important to consider the potential effects of secondary chemistry on the kinetics of the N(^2^D) + CH_2_CCH_2_ reaction. As the C(^3^P) + CH_2_CCH_2_ reaction is rapid at low temperature,^71^ leading to various C_4_H_4_ isomers and H atoms as the primary products,^72,73^ it competes with reactions 2a and 2b, lowering the production of N(^2^D) atoms in the flow. This can be seen clearly in Figure 4, where the peak N(^2^D) fluorescence signal of the experiments performed in the presence of CH_2_CCH_2_ (green triangles and blue squares) is significantly reduced compared to the experiment where CH_2_CCH_2_ is absent (red circles). A detailed analysis of the secondary reactions that could arise in such studies of the reactions of N(^2^D) atoms with unsaturated hydrocarbons has already been presented in previous work.^21,22^ In the present case, secondary reactions such as those between the CN product of reaction 2b in particular and CH_2_CCH_2_ lead to the formation of various unsaturated hydrocarbons containing a cyano group (such as cyanoallene)^74^ and H atoms, neither of which are expected to produce N(^2^D) atoms through subsequent reactions. Similarly, the reaction between the various C_4_H_4_ isomers that could be present in the flow and NO coreagent are not expected to lead to N(^2^D) production either, although no information on these processes could be found in the literature. Overall, considering the various secondary reactions that could be occurring in the flow, it seems unlikely that these processes would have an important influence on the accuracy of the present kinetic measurements.

**Figure 5:**
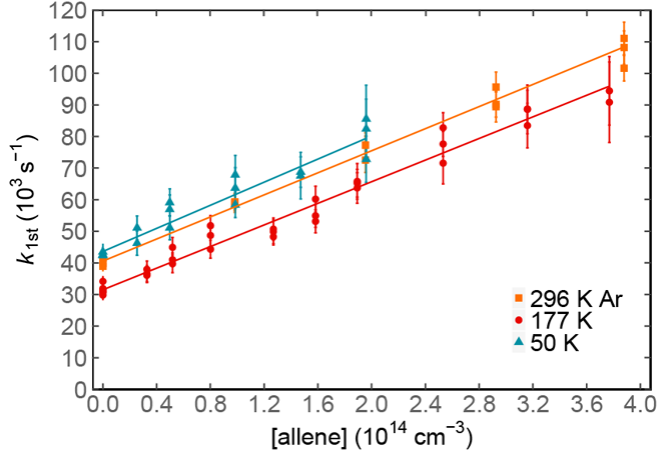
Measured pseudo-first-order rate constant as a function of [CH_2_CCH_2_]: (orange solid squares) data recorded at 296 K; (red solid circles) data recorded at 177 K; (blue solid triangles) data recorded at 50 K. Solid lines represent the weighted linear least-squares fits to the data. Errors on individual data points are shown at the level of a single standard deviation and are derived from the nonlinear fits of traces similar to those displayed in Figure 4.

Temporal profiles such as those shown in Figure 4 were recorded at a minimum of five different CH_2_CCH_2_ concentrations at each temperature. The values of the pseudo-first-order rate constants *k*_a_′, derived from fits to the data using eq 4, were then plotted as a function of the CH_2_CCH_2_ concentration to yield second-order plots such as those shown in Figure 5. Weighted fits to these data yielded the second-order rate constant from the slope.

The large *y*-axis intercept values of these plots (Figure 5) arise mostly from the reaction between N(^2^D) and NO, where NO is constant for any series of measurements at a given temperature. For example, at 177 K, considering [NO] = 4.1 × 10^14^ cm^–3^ and *k*_N(^2^D)+NO_(177 K) = 8 × 10^–11^ cm^3^ s^–1^,^48^ we obtain 32 800 s^–1^, a value that is seen to correspond well to the measured intercept in Figure 5. When a similar calculation is performed at 50 K however ([NO] = 4.2 × 10^14^ cm^–3^ and *k*_N(^2^D)+NO_(50 K) = 13 × 10^–11^ cm^3^ s^–1^),^48^ we obtain 54 600 s^–1^, somewhat larger than the measured intercept value here of 43 600 s^–1^. This discrepancy was also observed in our other recent studies of N(^2^D) reactions (N(^2^D) + C_2_H_2_^21^ and N(^2^D) + C_2_H_4_^22^), suggesting that the rate constant for *k*_N(^2^D)+NO_(50 K) might be slightly overestimated in the preliminary study of Nunez-Reyes et al.^48^

**Figure 6:**
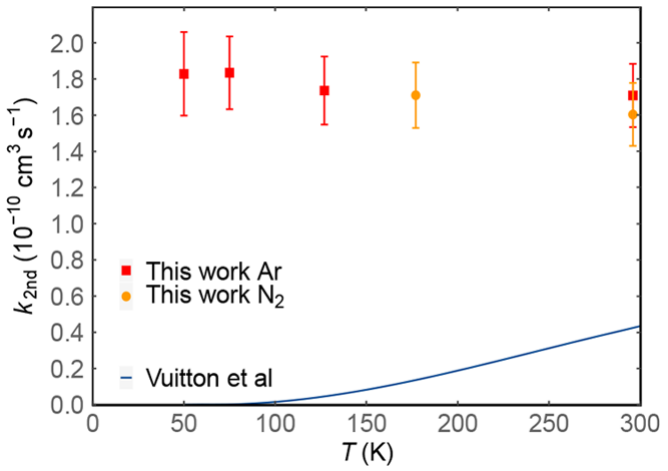
Second-order rate constants for the N(^2^D) + allene reaction as a function of temperature: (solid red squares) this work Ar carrier gas; (solid orange circles) this work N_2_ carrier gas. Solid dark blue line represents the current recommendation for the rate constants of the N(^2^D) + allene reaction used in the models of Vuitton et al.^5^ and Krasnopolsky.^75^

The measured second-order rate constants are plotted as a function of temperature in Figure 6, while these values are summarized in Table 1 alongside other relevant experimental parameters.

**Table 1:** Measured Second-Order Rate Constants for the N(^2^D) + CH_2_CCH_2_ Reaction

*T*/K	N^b^	[CH_2_CCH_2_]/10^14^ cm^–3^	[NO] /10^14^ cm^–3^	*k*_N(^2^D)+CH_2_CCH_2__/10^–11^ cm^3^ s^–1^
296 (N_2_)	22	0–4.9	6.4	(16.0 ± 1.7)^c^
296 (Ar)	18	0–4.8	6.3	(17.1 ± 1.8)
177 ± 2^a^	32	0–3.8	4.1	(17.1 ± 1.8)
127 ± 2	30	0–4.3	4.7	(17.4 ± 1.9)
75 ± 2	15	0–2.1	3.0	(18.3 ± 2.0)
50 ± 1	16	0–2.0	4.2	(18.3 ± 2.3)

a Uncertainties on the calculated temperatures represent the statistical (1σ) errors obtained from Pitot tube measurements of the impact pressure.

b Number of individual measurements.

c Uncertainties on the measured rate constants represent the combined statistical (1σ) and estimated systematic (10%) errors.

## 5 Theoretical Results

### 5.1 Potential Energy Surface

**Figure 7:**
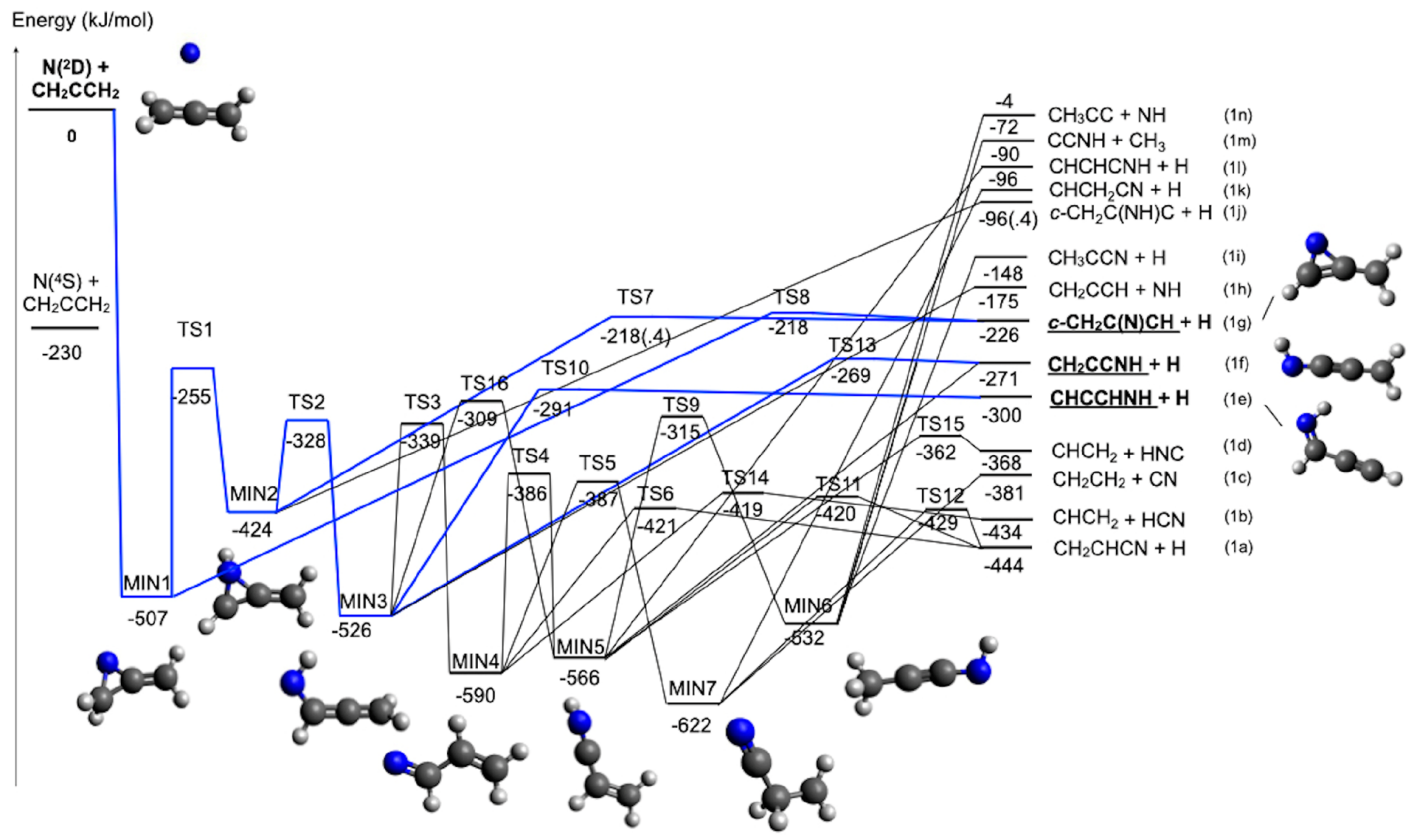
Schematic representation of the potential energy surface for the reaction N(^2^D) + CH_2_CCH_2_ with energies evaluated at the CCSD(T)/aug-cc-pVTZ level of theory (see text). Structures of the heavier coproducts from the three main product channels are shown as well as the structures of all intermediates. Blue lines indicate the main pathways leading to the underlined three (statistically predicted) main products.

The potential energy surface is shown in Figure 7. Seven minima have been identified (MIN1–7) linked by seven transition states (TS1, connecting MIN1 and MIN2; TS2, connecting MIN2 and MIN3; TS3, connecting MIN3 and MIN4; TS4, connecting MIN4 and MIN5; TS5, connecting MIN4 and MIN7; TS9, connecting MIN5 and MIN6; TS16, connecting MIN3 and MIN5). As expected, the initial step is represented by the barrierless attack of the nitrogen atom to one of the two double bonds of allene, leading to the formation of the cyclic intermediate MIN1, located at −507 kJ/mol (at the CCSD(T) level) with respect to the reactant asymptote. This first intermediate can directly dissociate to products via fission of a C–H bond, leading to c-CH_2_C(N)CH + H (channel 1g). An exit barrier of +8 kJ/mol with respect to the products asymptote (TS8) is present. Alternatively, MIN1 can isomerize to MIN2 (by overcoming TS1). MIN2 can also dissociate into the products associated with channel 1g by overcoming TS7 or into another set of products, that is, c-CH_2_C(NH)C + H (channel 1i) in a process without an exit barrier. Finally, by overcoming TS2 associated with ring opening, MIN2 can isomerize to MIN3, located 526 kJ/mol below the reactant energy asymptote. Two different H-loss channels have been identified starting from MIN3, one leading to the CHCCHNH isomer + H (channel 1e, via TS10) and one leading to the linear CH_2_CCNH isomer + H (channel 1f, via TS13). MIN3 can also decompose into propargyl radical + NH by fission of its C–N bond (channel 1h). Alternatively, MIN3 can isomerize to MIN4 by overcoming a barrier of +187 kJ/mol (TS3) or to MIN5 by overcoming a barrier of +217 kJ/mol (TS15).

**Figure 8:**
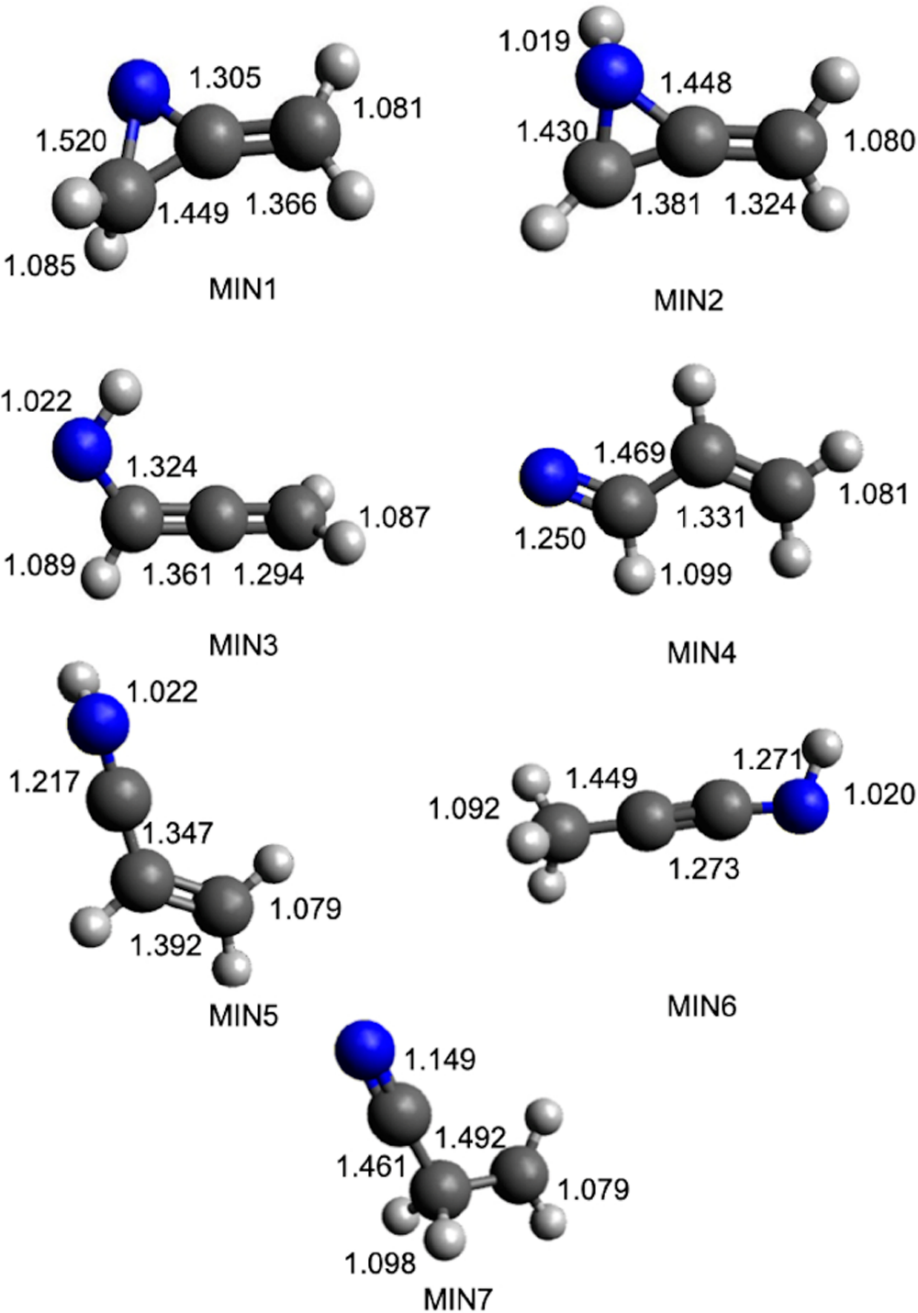
B3LYP-optimized geometries (in Angstroms) of the minima identified along the PES for the reaction N(^2^D) + CH_2_CCH_2_.

**Figure 9:**
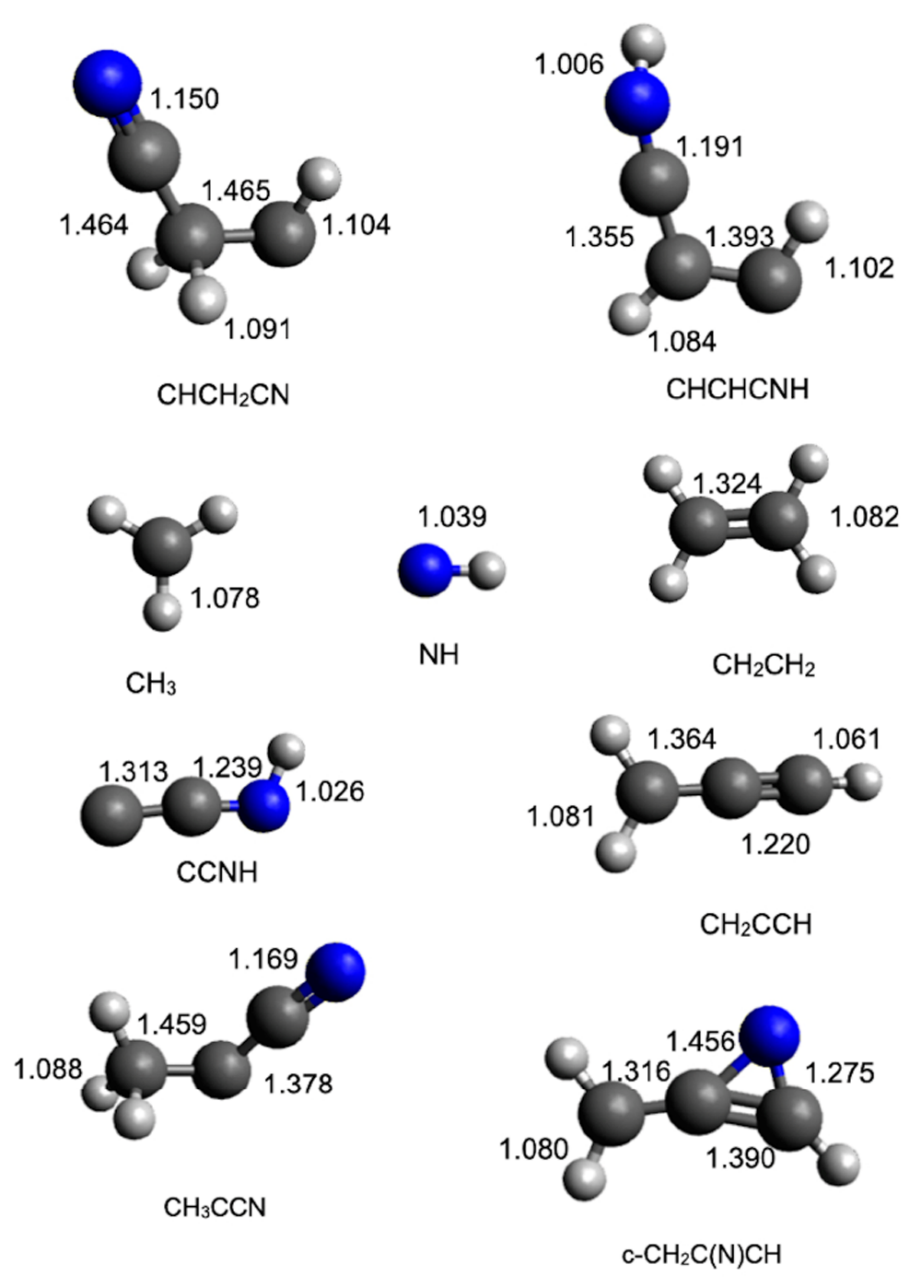
B3LYP-optimized geometries (in Angstroms) of the possible products identified along the PES for the reaction N(^2^D) + CH_2_CCH_2_.

**Figure 10:**
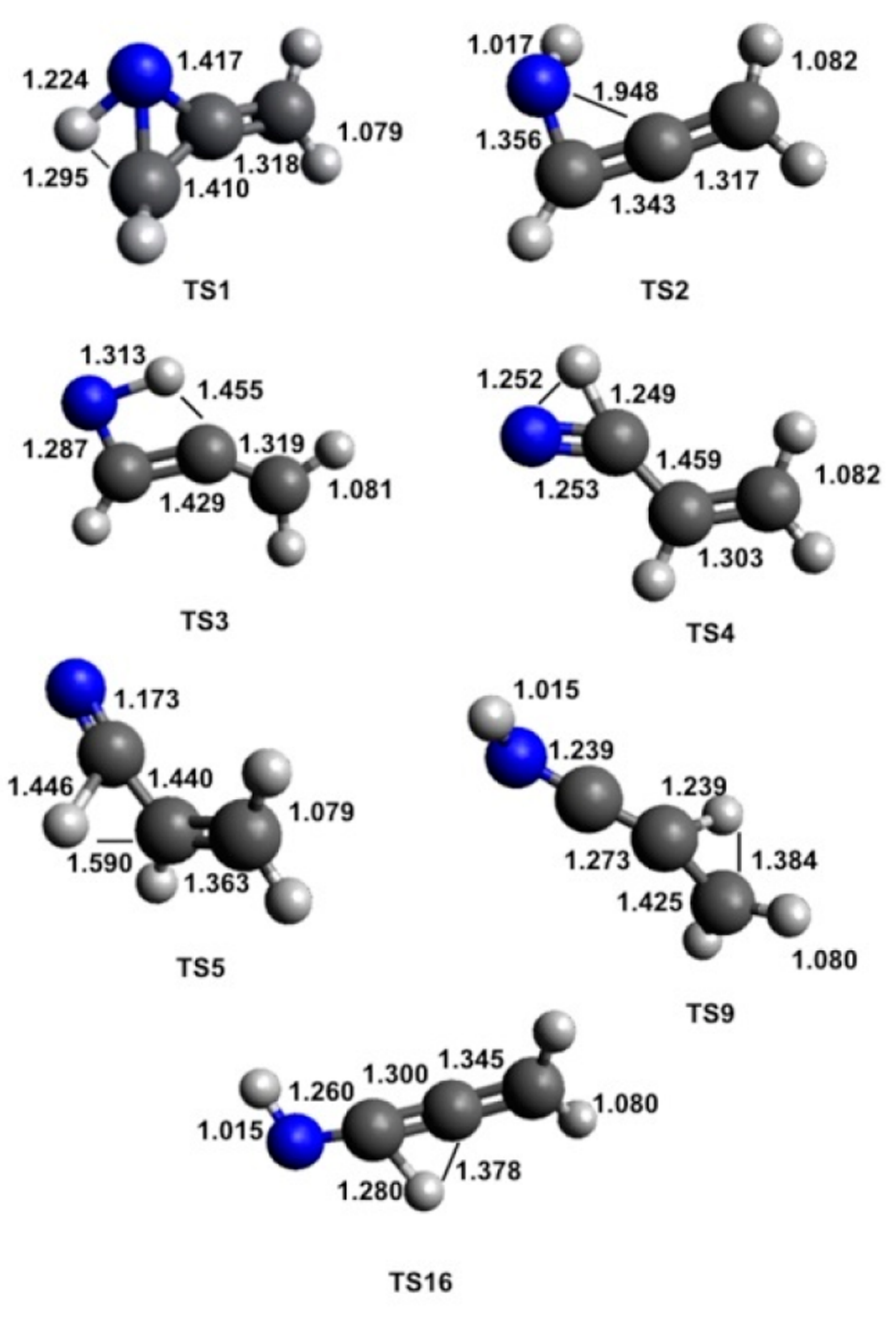
B3LYP-optimized geometries (in Angstroms) of the possible products identified along the PES for the reaction N(^2^D) + CH_2_CCH_2_.

**Figure 11:**
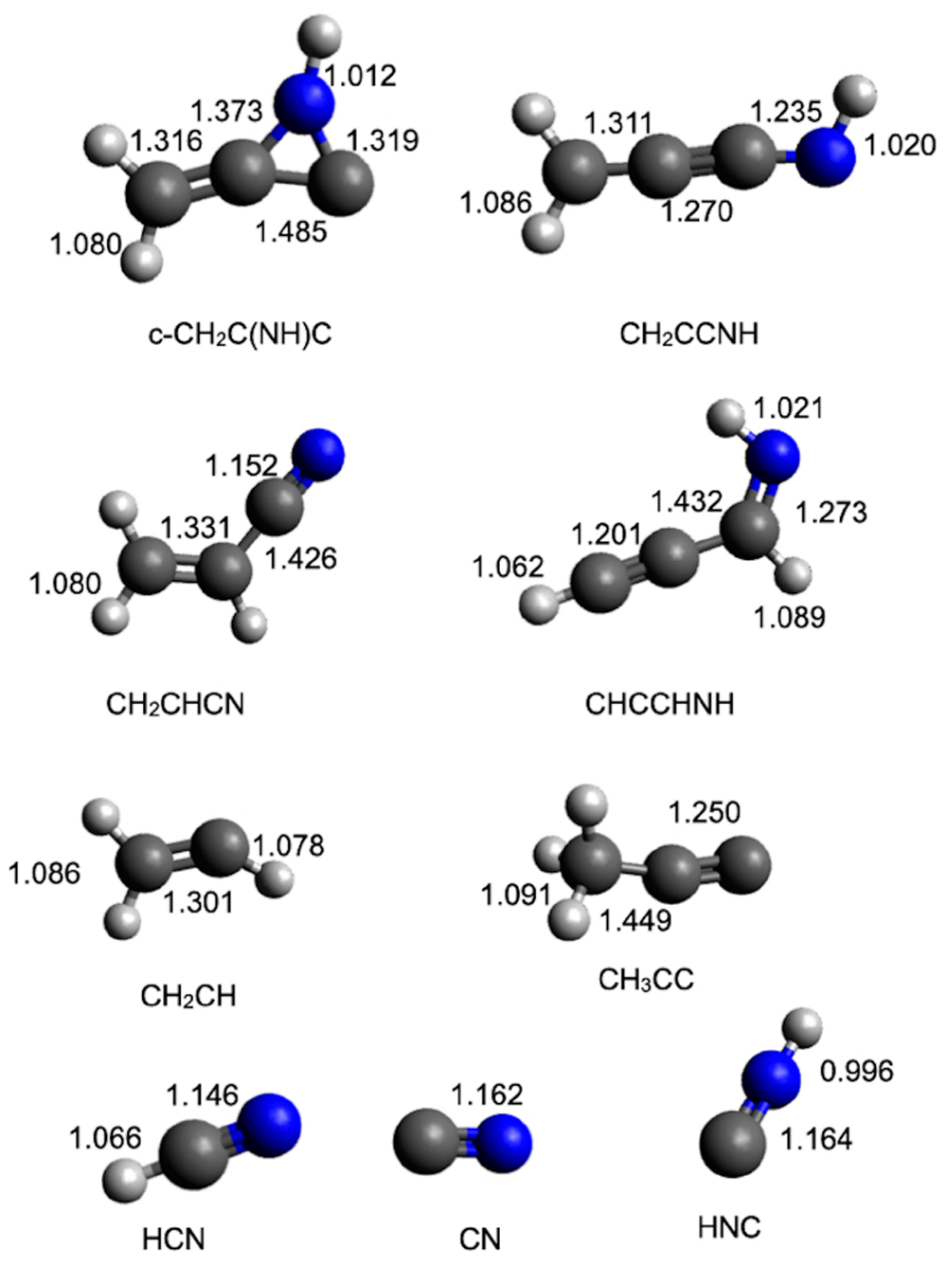
B3LYP-optimized geometries (in Angstroms) of the main transition states identified along the PES for the reaction N(^2^D) + CH_2_CCH_2_.

MIN4 can dissociate into HCN + CH_2_CH (channel 1b, exothermic by −434 kJ/mol) by overcoming the barrier associated with TS14 or into vinyl cyanide (CH_2_CHCN) + H (channel 1a, exothermic by −444 kJ/mol) by overcoming the barrier associated with TS6. MIN4 can also isomerize to MIN5 by overcoming a barrier of 204 kJ/mol (TS4). Once formed, MIN5 can dissociate into HNC + CH_2_CH (channel 1d, TS15) through the breaking of a C–C bond or into CH_2_CHCN + H (channel 1a, TS11), CH_2_CCNH + H (channel 1f), and CHCHCNH + H (channel 1k). Finally, MIN5 can isomerize to MIN6, located 532 kJ/mol below the energy of the reactants, overcoming a barrier of 248 kJ/mol (TS9). MIN6 can dissociate by breaking one of the C–H bonds, forming CH_2_CCNH + H (channel 1f) or CH_3_CCN + H (channel 1h). Alternatively, the breaking of a C–C bond can lead to the formation of CH_3_ together with the cofragment CCNH in a barrierless process. Fission of its C–N bond can also lead to CH_3_CC + NH in a nearly thermoneutral channel 1n. Finally, one last intermediate has been identified along the PES, MIN7, that can be formed starting from MIN4 after overcoming a barrier of 202 kJ/mol (TS5). MIN7 is the absolute minimum of the PES. The loss of a CN moiety can lead to the formation of ethylene (channel 1c, overall exothermic by 380 kJ/mol). In addition, a barrierless H-loss process can produce the fragment CHCH_2_CN (channel 1j) (located 96 kJ/mol below the reactant energy asymptote). Finally, by overcoming of a barrier of 194 kJ/mol (TS12), MIN7 can decompose into atomic hydrogen and vinyl cyanide (channel 1a). All of the identified stationary points lie below the energy level of the reactant asymptote. A schematic representation of the PES is shown in Figure 7, while in Table 2 the reaction enthalpies and barrier heights for each described step are reported, evaluated at the CCSD(T)/aug-cc-pVTZ level of theory considering the geometries optimized at the B3LYP/aug-cc-pVTZ level of theory. The geometries (distances in Angstroms) of the different minima and products identified along the PES together with the main saddle points optimized at the B3LYP/aug-cc-pVTZ level of theory are shown in Figures 8, 9, 10, and 11.

**Table 2:** Reaction Enthalpies and Barrier Heights (kJ/mol, 0 K) Computed at the CCSD(T)/aug-cc-pVTZ Level of Theory Considering the Geometries Obtained at the B3LYP/aug-cc-pVTZ Level for Dissociation and Isomerization Processes for the System N(^2^D) + CH_2_CCH_2_

	Δ*H*^0^_0_ (kJ/mol)	barrier heights (kJ/mol)
N(^2^D) + H_2_CCCH_2_ → MIN1	–507	
MIN1 → MIN2	83	252
MIN2 → MIN3	–102	96
MIN3 → MIN4	–63	188
MIN3 → MIN5	–39	218
MIN4 → MIN5	24	203
MIN4 → MIN7	–33	202
MIN5 → MIN6	34	251
MIN1 → H + c-CH_2_C(N)CH	281	289
MIN2 → H + c-CH_2_C(NH)C	328	
MIN2 → H + c-CH_2_C(N)CH	198	206
MIN3 → H + CHCCHNH	226	236
MIN3 → H + CH_2_CCNH	255	258
MIN3 → CH_2_CCH + NH	351	
MIN4 → HCN + CH_2_CH	155	170
MIN4 → H + CH_2_CHCN	145	169
MIN5 → H + CHCHCNH	476	
MIN5 → H + CH_2_CCNH	295	
MIN5 → HNC + CH_2_CH	198	204
MIN5 → H + CH_2_CHCN	122	146
MIN6 → CH_3_ + CCNH	461	
MIN6 → H + CH_3_CCN	391	
MIN6 → H + CH_2_CCNH	261	
MIN6 → CH_3_CC + NH	528	
MIN7 → H + CHCH_2_CN	527	
MIN7 → CN + CH_2_CH_2_	242	
MIN7 → H + CH_2_CHCN	178	194

**Figure 12:**
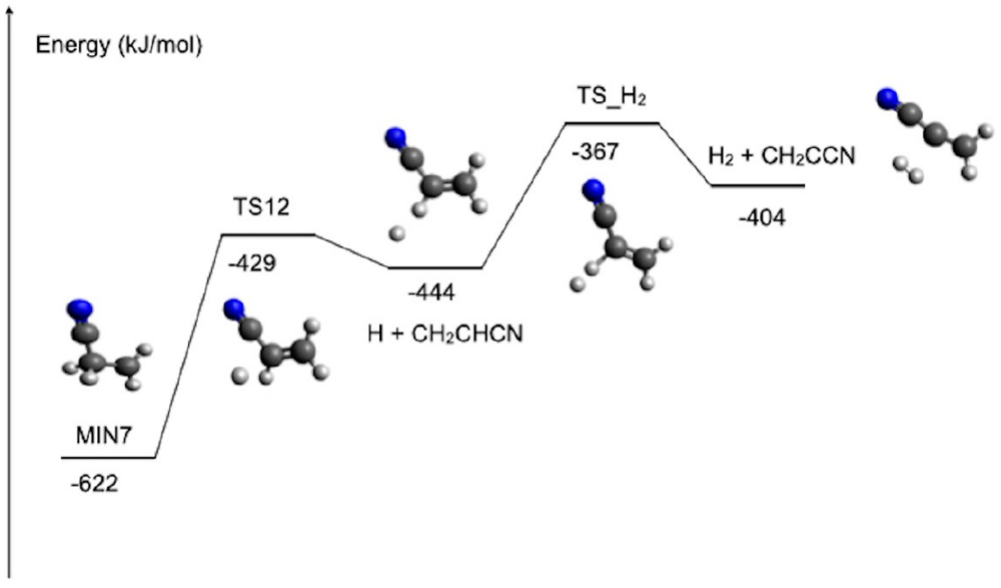
Schematic representation of the roaming mechanism leading to H_2_ and CH_2_CCN and originating from the interaction of the H atom emitted from MIN7 in conjunction with CH_2_CHCN formation (evaluated at the CCSD(T)/aug-cc-pVTZ level of theory, see text).

We thoroughly searched for a possible H_2_-elimination channel originating from one of the PES intermediates. We could not identify any possible route. However, we identified one path for H_2_ formation via a roaming mechanism (see Figure 12). The H atom emitted from MIN7 in conjunction with CH_2_CHCN formation can wander around and abstract the hydrogen atom of vinyl cyanide in the α position. The transition state, TS_H_2_, lies at an energy of −367 kJ/mol with respect to the reactants’ asymptote, that is +77 kJ/mol with respect to CH_2_CHCN + H.

We tried to verify whether N(^2^D) can insert into one of the C–H bonds of allene, but we have been unable to find this pathway. This is in line with previous studies of other systems, where N(^2^D) has shown the capability of inserting into C–H sigma bonds when carbon is characterized by sp^3^ hybridization (e.g., reactions with methane and ethane), but there are no known cases of insertion into C–H bonds when carbon is sp^2^ or sp hybridized (e.g., reactions with ethylene, acetylene, and benzene).

Finally, we characterized the H-abstraction mechanism. At the employed level of calculations, the relative transition state was very close in energy to the reactants. For this reason, we decided to further investigate this reaction at a higher level of accuracy. We optimized the geometry of the transition state and the reactants at the CCSD/cc-pVTZ level; at the same level of accuracy, we computed the vibrational frequencies and the zero-point energy (ZPE) correction. Then, we refined the energetics, evaluating the energy using a modified version^76,77^ of Martin’s extrapolation scheme^78^ in order to extrapolate the energies to the complete basis set (CBS) limit. The energies computed at the CCSD(T)/CBS level were then corrected with ZPE determined at the CCSD/cc-pVTZ level. At this very accurate level of calculation, the transition state for the H-abstraction reaction was computed to be 23.1 kJ/mol above the reactants, suggesting that this reaction cannot be relevant in astrochemical environments where the temperature is very low.

### 5.2 RRKM Branching Fractions

RRKM estimates of product branching fractions were performed considering the collision energy of the CMB experiment (33 kJ/mol) and for three different temperatures corresponding to the surface temperature of Titan (94 K), its stratospheric temperature (175 K), and room temperature (298 K). As can be seen from electronic structure calculations (Figure 7), the first step of the reaction between N(^2^D) and allene is the attack of the nitrogen atom to one of the two equivalent double bonds of allene, leading to formation of the c-H_2_CC(N)CH_2_ intermediate. This intermediate can directly dissociate into the products of channel 1g, or it can undergo several isomerization processes forming both cyclic and linear intermediates. All of the possible elementary processes, including back-isomerization, have been considered in the RRKM calculations to obtain the branching fractions, reported in Table 3. We recall that besides the relative energies of the TS and reactants, the density of states of both TSs and reactants are important factors that influence the values of rate constants (what would roughly correspond to the “entropy of activation”).

**Table 3:** Global Branching Fractions for All Possible Product Channels of the Reaction N(^2^D) + CH_2_CCH_2_

reaction channel	products	94 K	175 K	298 K	33 kJ/mol
1a	CH_2_CHCN + H	0.29%	0.29%	0.28%	0.25%
1b	C_2_H_3_ + HCN	0.43%	0.43%	0.42%	0.38%
1c	C_2_H_4_ + CN	0.01%	0.01%	0.01%	0.01%
1d	C_2_H_3_ + HNC	0.45%	0.45%	0.46%	0.46%
1e	CHCCHNH + H	11.43%	11.36%	11.22%	10.48%
1f	CH_2_CCNH + H	1.44%	1.44%	1.43%	1.38%
1g	c-CH_2_C(N)CH + H	85.95%	86.01%	86.17%	86.96%
1h	CH2CCH + NH	0.00%	0.01%	0.01%	0.08%
1i	CH_3_CCN + H	0.00%	0.00%	0.00%	0.00%
1j	c-CH_2_C(NH)C + H	0.00%	0.00%	0.00%	0.00%
1k	CHCH_2_CN + H	0.00%	0.00%	0.00%	0.00%
1l	CHCCHNH + H	0.00%	0.00%	0.00%	0.00%
1m	CH_3_ + CCNH	0.00%	0.00%	0.00%	0.00%
1n	CH_3_CC + NH	0.00%	0.00%	0.00%	0.00%

Under all of the considered conditions, the dominant channel is the one associated with the decomposition into c-CH_2_C(N)CH and an H atom (channel 1g) from the first intermediate. The second most important channel is that associated with the fission of a C–H bond from MIN3 leading to the formation of atomic hydrogen and propargyl imine (CHCCHNH) (channel 1e) with a branching fraction value of about 10%. Interestingly, the second dissociation process starting from MIN3 leading to CH_2_CCNH + H (channel 1f) does not seem to be competitive. Its branching fraction (also accessible from MIN5 and MIN6) is about 1%. Smaller contributions are associated with the formation routes of HCN and HNC starting from MIN4 and MIN5. The values of the branching fractions for the two pathways (channels 1b and 1d) are about 0.4%, while the formation of vinyl cyanide (channel 1a), which is the most exothermic channel in the potential energy surface, accompanied by the formation of H shows a value of branching fraction of about 0.3%. All of the other contributions can be considered negligible. Is should be noted that given the very small yield of channel 1a, the roaming mechanism that could lead to the formation of H_2_ and CH_2_CCN is not a significant reaction channel. This is in line with the lack of observation of reactive scattering associated with the heavy coproduct at *m*/*z* = 52.

Notably, there is little dependence of the products BFs on the energy available to the system.

## 6 Discussion

As already mentioned, within the sensitivity of our CMB experiments, we did not observe reactive signals associated with HCN, HNC, CN, and CH_3_ products, thus indicating that their BFs are smaller than 5–10%. Instead, the experimental data clearly demonstrate that one or more H-displacement channels are occurring. According to our electronic structure calculations, eight isomers with gross formula C_3_H_3_N can be formed (see sections 3.1 and 5.1). A satisfactory fit of the LAB angular and TOF distributions was achieved by using a single set of CM functions, which implies that our data are not sensitive enough to allow disentangling the possible different contributions originating from more than one channel to the signal at the same mass.

The CM product angular distribution provides us with some information on the reaction mechanism, i.e., its characteristics indicate whether the reaction is direct (that is, it occurs on the time scale of molecular vibrations) or proceeds via the formation of a long-lived complex intermediate^70,79,80^ (that is, it occurs within the time necessary for several rotations). Furthermore, the product translational energy distribution is determined by the characteristics of the PES and provide us information on the product energy partitioning between translational and internal degrees of freedom. As already noted in section 4.1, the backward–forward symmetric *T*(θ) (Figure 3, top) indicates that the formation of C_3_H_3_N isomeric products proceeds through a long-lived complex mechanism.^70,79,80^ This is fully supported by the reaction PES, which is characterized by bound intermediates associated with deep wells along all possible reaction pathways (see Figure 7). As noted in section 4.1, the shape of the best-fit *T*(θ) (and of the functions that still allow an acceptable fit of the experimental data) are in line with the formation of a long-lived complex. Therefore, the expected randomization of the available energy justifies the statistical approach underlying the RRKM method that we used to derive the product branching fractions from the characteristics of the PES.

The energy release, revealed by the shape of the *P*(*E*′_T_) (Figure 3, bottom), provides us with a criterion (through the energy conservation rule^70^) to establish which channels are responsible for the experimental data. The *P*(*E*′_T_) cutoff defines the maximum available energy of the products, and the vertical lines represented in Figure 3 (bottom) indicate the total available energy, *E*_tot_, for the four most exothermic isomeric channels of interest. Clearly, the best-fit *P*(*E*′_T_) is consistent with the energetics of four (out of the eight possible) H-displacement channels, namely, channels 1a, 1e, 1f, and 1g. In contrast, the other H-displacement channels 1i–1l can only give a minor contribution. Finally, the cutoff of 335 ± 20 kJ/mol indicates that the most exothermic channel 1a is probably minor with respect to channels 1e, 1f, and 1g.

As can be seen from Table 3, according to RRKM predictions, the three main reaction channels are all H-displacement channels 1e, 1f, 1g with channel 1g, leading to formation of c-CH_2_C(N)CH, being by far the dominant reaction channel (BF = 87%) under all conditions. The intermediates that can lead to the c-CH_2_C(N)CH + H channel are MIN1 and MIN2, but the main pathway is the one associated with MIN1. The competition between dissociation into c-CH_2_C(N)CH + H and isomerization to MIN2 is much more in favor of the dissociation despite the fact the barrier associated with product formation (TS8) is 37 kJ/mol higher in energy with respect to the barrier associated with isomerization (TS1). Notably, the most exothermic channel, leading to vinyl cyanide (CH_2_CHCN) + H (channel 1a), is theoretically predicted to be negligible (BF = 0.25%) because only a small portion of the reactive flux that reaches MIN3 and MIN3 preferentially dissociates into channel 1e (BF = 10.5%) via TS10 (at −291 kJ/mol respect to reagents) rather than isomerizes to MIN4 (via TS3 at −339 kJ/mol), which is the precursor of channel 1a.

From the product translation energy distribution, we derived the average product translational energy released. If we refer to channel 1g (which has been indicated as the most important one by RRKM estimates of BF), the fraction of product translational excitation, ⟨*f*′_T_⟩, is about 0.45 of the total available energy (*E*_tot_ for each product channel is indicated by a vertical line in Figure 3, bottom). The ⟨*f*′_T_⟩ reduces to 0.35 if we refer to the energetics of channel 1e. These values suggest relatively tight exit transition states (TS8, TS13, and TS10 in Figure 1) and the formation of highly internally excited products.

As far as the kinetics of the title reaction is concerned, in common with the reactions of N(^2^D) atoms with other unsaturated hydrocarbons such as C_2_H_2_ and C_2_H_4_, it can be seen that the rate constants for the N(^2^D) + allene reaction are large and independent of temperature, considering the associated experimental uncertainties. Moreover, the carrier gas itself seems to have little or no influence on the measured rates. Consequently, we recommend a temperature-independent value for the rate constant of 1.7 ± 0.2 × 10^–10^ cm^3^ s^–1^ over the 50–300 K range. It should be noted here that the measured rate constant is a sum of reactive and nonreactive “quenching” losses through collisions with CH_2_CCH_2_. Nevertheless, given the barrierless nature of the reaction and the absence of any substantial submerged barriers over the PES, it is unlikely that quenching plays an important role here.

In Figure 6, we also show the currently recommended values for the rate constants of the N(^2^D) + allene reaction as employed in recent photochemical models of Titan’s atmosphere by Krasnopolsky^75^ and Vuitton et al.^5^ These values were estimated by adopting the Arrhenius parameters derived by Sato et al.^24^ during their kinetic investigation of the N(^2^D) + C_2_H_4_ reaction in a limited range of temperature between 230 and 292 K. We recall that the N(^2^D) + C_2_H_4_ reaction has been recently investigated by some of the present authors^22^ in the temperature range of interest for Titan, and the values of the rate coefficients were seen to slightly increase with decreasing temperature (the inverse trend in the case of the data by Sato et al.^24^).

Assuming an average temperature of 170 K for the atmosphere of Titan, the rate constant of 1.2 × 10^–11^ cm^3^ s^–1^ (currently recommended in the photochemical models of Titan) is 14 times smaller than the temperature-independent value measured during the present work.

### 6.1 Comparison with N(^2^D) + CH_3_CCH (methylacetylene)

It is of interest to compare the reaction dynamics of N(^2^D) + allene with that of the isomeric reaction N(^2^D) + methylacetylene recently studied in our laboratory at a comparable *E*_c_.^18^ Methylacetylene and allene are structural isomers that are normally not distinguished in astrochemical or photochemical models, being simply indicated with their gross formula C_3_H_4_. However, it has been already noted that their formation and destruction routes are indeed different in many cases.^81^ In the photochemical model of Titan, methylacetylene and allene are formed through various reactions including
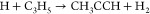
5a
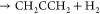
5bfor which a recent estimate of the rate coefficient in the high-pressure limit is 3.4 × 10^–10^ cm^3^ s^–1^.^82^ However, the product branching fraction of reaction 5 has never been derived. In the model by Lavvas et al.,^83^ the main formation route of both methylacetylene and allene is as follows
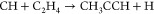
6a
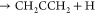
6ba fast process where CH_3_CCH and CH_2_CCH_2_ are again assumed to be formed with the same yield. However, experimental studies have shown that the formation of allene was favored in this reaction.^84,85^

Concerning the reactivity of CH_3_CCH/CH_2_CCH_2_, while it is true that their bimolecular reactions are often characterized by similar rate coefficients, the reaction products can be very different. For instance, the dominant product channels of the O(^3^P) + methylacetylene and O(^3^P) + allene isomeric reactions lead in both cases to CO formation, but the coproducts are singlet ethylidene (^1^CH_3_CH) and singlet ethylene (CH_2_CH_2_), respectively.^86−88^ Another recent example comes from the reaction with the BO radical, where the reaction with methylacetylene features the CH_3_-elimination channel as being largely dominant when the exclusive channel for the reaction with allene leads to the formation of CH_2_CCHBO in a H-displacement channel.^89^

The case of the reactions with N(^2^D) are in the same vein. In the reaction with methylacetylene, the main channels are those leading to CH_2_NH + C_2_H (BF ca. 40%), c-C(N)CH + CH_3_ (BF 28–32%), and CH_2_CHCN + H (BF 12–16%).^14^ Therefore, there are significant differences even though the PESs for the two reactions share some common intermediates. Such a different behavior can be explained by the fact that according to our RRKM calculations, direct decomposition of the first addition intermediate MIN1 is the dominant process in the case of the title reaction, and therefore, the isomerization steps that lead to the formation of the other channels are not competitive. The same is true also for the N(^2^D) + methylacetylene reaction^14^ that, however, features two possible barrierless attacks leading to two different addition intermediates (one corresponding to bridge addition and one corresponding to the N insertion into one of the C–H bonds of the methyl group). Furthermore, in the case of the N(^2^D) + CH_3_CCH reaction, there are only three isomerization steps which are necessary to access the most exothermic channel leading to vinyl cyanide and H, all of them favored with respect to their competitive processes, while for the case of the title reaction, four isomerization steps are necessary, starting from the first intermediate, with alternative pathways being more feasible.

## 7 Implication for the Atmosphere of Titan

To examine the influence of the present measurements on the chemistry of Titan’s atmosphere, we included the N(^2^D) + allene reaction in a 1D photochemical model described by Dobrijevic et al.,^90^ which treats the chemistry of neutrals and cations (we do not consider anions in this study as they play a very minor role), and the coupling between them from the lower atmosphere to the ionosphere. Two different simulations were performed during this investigation. The first one neglected the N(^2^D) + allene reaction, which was the case in the previous model. For the second one, we included the N(^2^D) + allene reaction using the rate constants (1.7 ± 0.2 × 10^–10^ cm^3^ s^–1^) and branching fractions (slightly simplified) determined in this study (see Table 3). As the N(^2^D) + allene reaction produces two new species, CHCCHNH and c-CH_2_C(N)CH, we developed a chemical network to describe these species by considering their most important reactions. For the reactions with barriers, the barrier heights have been calculated theoretically with the Gaussian program^65^ using DFT associated with the M06-2X functional and the aug-cc-pVTZ basis set. We also computed the absorption spectra of these species by calculating the energy of the excited states and the oscillator strengths of the transitions from the ground state using the EOM-CCSD(T)/aug-cc-pVTZ method. The main effect of the inclusion of the N(^2^D) + allene reaction is the production of the two new species HCCCHNH and c-CH_2_C(N)CH. Indeed, inclusion of the N(^2^D) + allene reaction has only a minor effect on the allene concentration, decreasing its abundance by 8% at 1200 km but very little at low altitude. The integrated column density over the whole atmosphere is only slightly affected (less than 1% decrease). Note that this effect would be even smaller using the rate constant expression recommended by Vuitton et al.^5^ The new species produced by the N(^2^D) + allene reaction (HCCCHNH and c-CH_2_C(N)CH) are relatively abundant in the upper atmosphere where N(^2^D) is produced. However, their calculated relative abundance are 100 times lower than that of HNC, a species with a similar abundance profile. These low relative abundance limit their possible detection by microwave spectroscopy despite their relatively strong dipole moments calculated around 2.2–2.6 D in both cases. In contrast, the high estimated reactivities of HCCCHNH and c-CH_2_C(N)CH with atomic hydrogen and their photodissociation cross sections in the near UV considerably limit their simulated abundance at low altitudes, which would prevent their detection by IR spectroscopy.

We recall that the N(^2^D) + allene reaction is considered to produce C_3_H_3_N (C_2_H_3_CN in fact) in the modeling study by Vuitton et al.,^5^ while the channel leading to C_2_H_3_CN is negligible according to the present CMB results even if C_2_H_3_CN is more thermodynamically stable than its isomers HCCCHNH and c-CH_2_CNCH.

## 8 Conclusions

The N(^2^D) reaction with allene was investigated by the CMB technique with mass spectrometric detection at a collision energy of 33 kJ/mol coupled with electronic structure calculations of the underlying potential energy surface. The angular and TOF distributions of C_3_H_3_N products in the LAB frame along with the derived CM best-fit functions suggest that the reaction mechanism features the formation of one or more C_3_H_4_N intermediates with lifetimes longer than their rotational periods. The translational energy distribution reveals that C_3_H_3_N products are internally (ro-vibrationally) excited and that the most exothermic of all possible H-forming channels, namely, cyanoethylene (acrylonitrile or vinylcyanide) + H, is formed with low probability, while other isomers of acrylonitrile are important. Synergistic RRKM statistical calculations on the doublet C_3_H_4_N PES of product distributions and branching fractions corroborate and complement our findings for the H-displacement channels and provide a more complete picture of the overall reaction mechanism with up to 14 competing product channels being open and for which product BFs are calculated as a function of energy. Of these 14 channels, 9 feature a BF < 1%. Our calculations show that this reaction is initiated by the barrierless addition of the N(^2^D) atom to the double bonds of CH_2_CCH_2_ forming a cyclic adduct complex c-CH_2_C(N)CH_2_ (MIN1). By the breaking of the C–H bond, this intermediate can directly dissociate predominantly to c-CH_2_C(N)CH + H with a predicted BF of about 87% or competitively isomerize to MIN2 and successively to a variety of linear complexes (from MIN3 to MIN7) of which MIN3 dominates, by C–H bond cleavage, to the second and third most important product channels CHCCHNH + H with BF ≈ 10% and CH_2_CCNH + H with BF ≈ 1.4%, respectively. All other exothermic channels contribute for well less than 1% (Table 3).

Our studies indicate that the reaction of N(^2^D) with CH_2_CCH_2_, in contrast to the reaction of N(^2^D) with the isomer CH_3_CCH,^14^ is not a potential pathway to produce, in the conditions of the atmosphere of Titan, methanimine (CH_2_NH), c-C(N)CH, and acrylonitrile (CH_2_CHCN) in the gas phase but rather, via H displacement, predominantly c-CH_2_C(N)CH, CHCCHNH, and CH_2_CCNH.

Kinetic experiments, from room temperature down to 50 K indicated that the rate constants for the N(^2^D) + allene reaction are large and independent of temperature, considering the associated experimental uncertainties. We therefore recommend a temperature-independent value for the rate constant of 1.7 ± 0.2 × 10^–10^ cm^3^ s^–1^ over the 50–300 K range. Assuming an average temperature of 170 K for the atmosphere of Titan, this value is 14 times larger than the currently recommended^5,6^ rate constant of 1.2 × 10^–11^ cm^3^ s^–1^. While the reaction between N(^2^D) and allene has a negligible effect on the simulated abundance of C_2_H_3_CN, HCCCHNH and c-CH_2_C(N)CH are predicted to be relatively abundant in the upper atmosphere where N(^2^D) is produced. These species might react further with other molecules acting as precursors for nitriles (C_2_N_2_, C_3_N) or other more complex organic molecules containing a CN bond.
